# Rapid Authentication and Detection of Olive Oil Adulteration Using Laser-Induced Breakdown Spectroscopy

**DOI:** 10.3390/molecules28247960

**Published:** 2023-12-05

**Authors:** Eleni Nanou, Nefeli Pliatsika, Stelios Couris

**Affiliations:** 1 Department of Physics, University of Patras, 26504 Patras, Greece; e.nanou@iceht.forth.gr (E.N.); n.pliatsika@iceht.forth.gr (N.P.); 2 Institute of Chemical Engineering Sciences (ICE-HT), Foundation for Research and Technology-Hellas (FORTH), 26504 Patras, Greece

**Keywords:** laser-induced breakdown spectroscopy, LIBS, olive oil, EVOO, adulteration, authenticity, machine learning

## Abstract

The adulteration of olive oil is a crucial matter for food safety authorities, global organizations, and consumers. To guarantee olive oil authenticity, the European Union (EU) has promoted the labeling of olive oils with the indices of Protected Designation of Origin (PDO) and Protected Geographical Identification (PGI), while food security agencies are also interested in newly emerging technologies capable of operating reliably, fast, and in real-time, either in situ or remotely, for quality control. Among the proposed methods, photonic technologies appear to be suitable and promising for dealing with this issue. In this regard, a laser-based technique, namely, Laser-Induced Breakdown Spectroscopy (LIBS), assisted via machine learning tools, is proposed for the real-time detection of olive oil adulteration with lower-quality oils (i.e., pomace, soybean, sunflower, and corn oils). The results of the present work demonstrate the high efficiency and potential of the LIBS technique for the rapid detection of olive oil adulteration and the detection of adulterants.

## 1. Introduction

Mediterranean countries are commonly known for their extensive cultivation of olive trees and large olive oil production. According to the International Olive Council (IOC), Spain is the largest producer and exporter of olive oil in the world, followed by Italy and Greece, with Tunisia, Turkey, Morocco, and Portugal also being significant olive-oil-producing and exporting countries [1]. The favorable environmental conditions (climate, temperature, humidity, soil, etc.) that prevail in the countries of the Mediterranean Basin foster olive oil production and contribute to the production of high-quality olive oil, distinguished by its unique color, flavor, taste, and smell.

At the pinnacle of the olive oil hierarchy list, extra virgin olive oil (EVOO) is the premier olive oil, followed by virgin olive oil (VOO), along with regular olive oil (OO), refined olive oil (ROO), and pomace olive oil (POO) [2]. EVOO and VOO are the most consumed ones by those who follow the Mediterranean diet, owing to their various health benefits. However, since the procedures for the extraction of olive oil are notably demanding, especially apropos of EVOOs and VOOs, they have an impact on the price of these products in the market [3]. The large price gap between EVOO/VOO and other types of olive oils or non-olive oils is one of the main reasons for fraudulent activities to occur.

EVOO and VOO are generally susceptible to adulteration with lower-quality oils, including soybean, sunflower, sesame, canola, and corn oil, among others. In addition, pomace oil, which is extracted from the residue of olive oil fruits, is one of the commonly used adulterants. To prevent fraud and secure the authenticity of olive oil, authorities have established certification procedures, such as the European Union’s indices of Protected Designation of Origin (PDO) and Protected Geographical Identification (PGI) [4], which guarantee that they have been prepared according to the highest quality standards and have characteristics that make them unique, such as certain olive varieties that are closely linked to a well-defined region and a time-honored tradition. Yet, these seals are not enough, and illegal practices still occur due to economic motives, raising the concern of food authorities and agencies for fraud traceability. In this regard, additional efforts have been directed toward developing new strategies for the protection of olive oil quality, particularly new cutting-edge technologies.

Among the proposed methods, photonic technologies and spectroscopic methods, which share a unique set of advantages (e.g., real-time, online, in situ, and/or remote operation; minimal or no sample preparation; and non-invasiveness), appear to be suitable for the detection of olive oil adulteration. Thus, Raman spectroscopy, Near-Infrared (NIR) spectroscopy, Fourier Transform Infrared spectroscopy (FTIR), and Laser-Induced Fluorescence spectroscopy (LIF) have been applied for food quality and safety issues and the detection of olive oil adulteration. Georgouli et al. [5] used Raman spectroscopy and FTIR to detect EVOOs’ adulteration with hazelnut oil by studying samples with different levels of adulteration (e.g., from 1 to 90%) and employing different machine learning approaches (Continuous Locality Preserving Projections (CLPPs), k-Nearest Neighbors (k-NNs), etc.). They reported quite high discrimination accuracies. Mu et al. [6] applied LIF and different chemometric tools (e.g., Principal Component Analysis (PCA), Partial Least Squares Regression (PLSR), Support Vector Machines (SVMs), and Artificial Neural Networks (ANNs)) to distinguish EVOOs from EVOOs adulterated with different percentages of peanut oil and rapeseed oil samples (in the range of 2.5 to 50%). In this study, classification accuracies up to 100% were attained using SVM and ANN algorithms, while a correlation coefficient value (R^2^) of 0.99 was obtained for the PLSR model. Meng et al. [7] performed a similar investigation by means of FTIR, VIS-NIR, and excitation–emission matrix fluorescence spectroscopies, studying various olive oils adulterated with soybean oil at percentages from 5 to 50%. For the analysis of the data, PCA/multi-way PCA and Partial Least Squares Discriminant Analysis (PLS-DA) were employed, providing successful results, as well.

More recently, Laser-Induced Breakdown Spectroscopy (LIBS) has also been proposed and applied for the authentication of olive oil. LIBS is a relatively simple laser-based technique in which a strong enough laser beam is focused on a sample, inducing dielectric breakdown, i.e., producing a micro-plasma, which consists of excited atoms, ions, molecules or fragments of molecular species, and electrons. The excited species thus produced can emit characteristic radiations, i.e., a “fingerprint” of the sample, thus providing valuable qualitative and quantitative information about the sample’s elemental composition. Besides the experimental simplicity of LIBS, its main attributes are minimum or no sample preparation and its ability to be applied to samples of all states (solid, liquid, and gas), whether dielectric or not [8,9]; it can also provide rapid, in-situ, online, and real-time measurements. Due to these advantages, LIBS has been proposed for a wide range of applications, such as industrial [10], environmental/recycling [11,12], cultural heritage [13,14], food analysis [15,16], etc.

Regarding olive oil authenticity and traceability, LIBS has been successfully applied for the detection of olive oil adulteration. Specifically, Caceres et al. [17] used LIBS for the detection of adulteration in olive oils mixed with different commercially available oils (i.e., sunflower seed, corn, and hazelnut oil) employing a neural network algorithm. They reported high classification accuracies, up to 95%. In a similar work conducted by Bellou et al. [18], the discrimination of pure olive oil samples from samples adulterated with pomace oil was investigated using LIBS, Principal Component Analysis (PCA), and Linear Discriminant Analysis (LDA). Again, high classification accuracies were obtained. In another work, Kongbonga et al. [19] employed LIBS to study the C_2_ Swan band in various olive and vegetable oils (e.g., refined sunflower, corn, and palm oils, as well as crude palm oil) and correlated it to the content of saturated fatty acids.

Furthermore, LIBS has also been employed for the recognition of olive oil’s geographical and cultivar origin. Gazeli et al. [20] and Gyftokostas et al. [21] used LIBS, aided via machine learning, to discriminate olive oil samples from different regions of Crete, Greece. In both studies, high classification accuracies were obtained, i.e., from 90 to 99% in the former and up to 94% in the latter. In addition, Gyftokostas et al. [22] employed LIBS and chemometrics to identify the geographical origin of olive oils from three regions of Greece, namely, Crete, Peloponnese, and Lesvos. The analysis of the spectroscopic data via machine learning tools resulted in accuracies up to 100%. In an extension of this work, Gyftokostas et al. [23], using LIBS and UV-Vis-NIR absorption spectroscopy and chemometrics, studied the discrimination of several olive oils and binary mixes of them from different regions in Greece based on their geographical origin. The algorithmic models applied were evaluated for their efficiency, resulting in predictive accuracies up to 99%. Similarly, Stefas et al. [24] used the fusion of the spectroscopic data from LIBS and UV-Vis-NIR absorption to discriminate olive oils in terms of cultivar origin. In all the above studies, remarkably successful results were obtained, and discrimination accuracies from 82% to 100% were reported.

The present work reports on the application of the LIBS technique, assisted via machine learning, for the detection of the adulteration of EVOOs with various lower-quality oils, often used in fraudulent activities. In this view, 40 Greek EVOOs from four regions of Greece traditionally known for the quality and special characteristics of their olive oil, namely, Crete, Lesvos, Kalamata, and Achaia, were mixed at different concentrations, with different commercially available oils of lower quality, namely, pomace, corn, soybean, and sunflower oil, and were studied using LIBS. The obtained spectroscopic data (i.e., LIBS spectra) were analyzed via different machine learning algorithms, i.e., Principal Component Analysis (PCA), Linear Discriminant Analysis (LDA), Support Vector Machines (SVMs), Logistic Regression (LR), and Gradient Boosting (GB), and were assessed for their efficiency to identify pure EVOOs from adulterated ones. Then, corresponding statistical models were constructed and used for the discrimination of the pure EVOOs from the adulterated ones and the identification of the adulterant used. The effectiveness and robustness of the models for successful predictions were evaluated via internal and external validation procedures. In all cases, high classification and prediction accuracies were obtained. The present findings demonstrate the ability of the LIBS technique, assisted via machine learning, for the fast and reliable real-time detection of adulteration in olive oil.

## 2. Results and Discussion

In the present work, the potential of the LIBS technique, aided via machine learning, to detect EVOOs’ adulteration was initially investigated. For this purpose, the LIBS spectroscopic data were divided into two classes: the class of pure EVOOs and the class of all mixtures with the four different lower-quality oils (i.e., corn, pomace, soybean, and sunflower oil). The first class comprised EVOOs from different geographical regions (i.e., 40 samples), and the second one comprised all the prepared binary mixtures of EVOO/lower-quality oil, with concentrations ranging from 10 to 90%, with a step of 10% (i.e., 144 samples in total). Additionally, the possibility of identifying the adulterant used was also examined. For this goal, the mixtures of EVOOs with each adulterant were considered as four separate classes (i.e., 36 corn-, 36 pomace-, 36 soybean-, and 36 sunflower-mixed samples), and all EVOOs as one class. Next, in a different approach, concerning the identification of the type of adulterant used, the EVOOs of each geographical region (i.e., Crete, Lesvos, Kalamata, and Achaia) and their mixtures with each one of the four adulterants (i.e., 9 samples for each adulterant/class, 46 samples in total) were considered separately and were analyzed.

### 2.1. Discrimination of Pure EVOOs from Adulterated EVOOs

First, for the analysis of the LIBS spectroscopic data of all EVOOs (considered as one class) and all adulterated EVOO samples (considered as another class), the PCA algorithm was applied for dimensionality reduction purposes. From the optimization of the algorithm, it was concluded that 60 Principal Components (PCs) were enough to maintain most of the initial spectral information. Then, the preprocessed data were introduced into the LDA, SVM, LR, and GB algorithms, and the corresponding predictive models were constructed. The obtained results were remarkable, as high classification and predictive accuracies were attained. Specifically, classification accuracies up to 100% were obtained using the LDA, SMV, and LR algorithms, as well as 99.7% using the GB one (see Table 1). More importantly, predictive accuracies as high as 100% for the LDA, SMV, and LR algorithms, as well as 99.8% for the GB one (see Table 1), were reached, demonstrating the efficiency of the models in distinguishing pure EVOO samples from adulterated ones. In more detail, this is also shown by the corresponding confusion matrices presented in Table 1, where it is shown that the EVOOs and the adulterated ones were all correctly classified in their respective class, except one misclassification occurred using the GB algorithm.

### 2.2. Identification of the Type of Adulterant (i.e., Corn, Pomace, Soybean, and Sunflower Oils)

**Figure 1:**
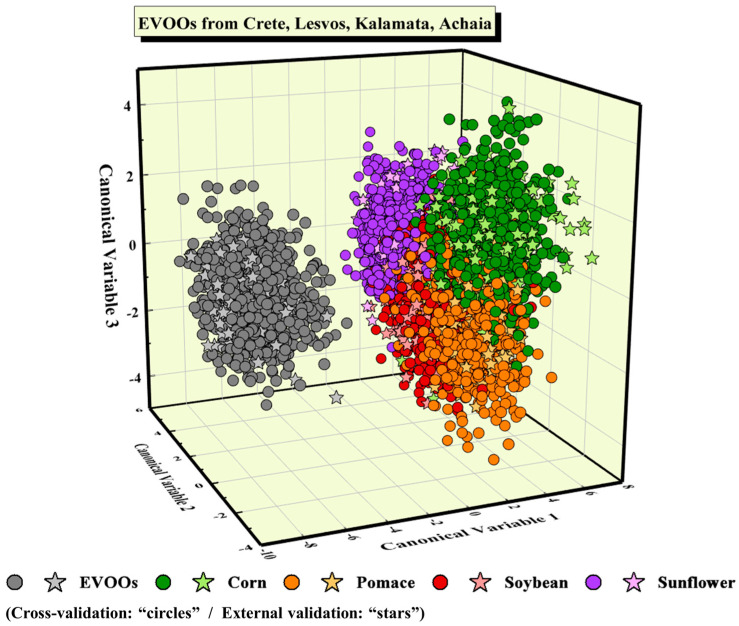
LDA plot showing the classification of all EVOOs and their mixtures with corn, pomace, soybean, and sunflower oils.

Next, the possibility of identifying the type of adulterant was studied; all EVOOs (i.e., 40 samples in total) were considered as one class, with the adulterated ones (i.e., 36 samples per adulterant) forming four classes. Again, the PCA algorithm was employed for dimensionality reduction, and the number of PCs required to construct robust predictive models was determined. It resulted that 130 PCs were adequate. Then, the PCA-pre-treated LIBS spectroscopic data were analyzed using the LDA algorithm. It is useful, at this point, to note that the LDA algorithm, apart from the construction of a predictive model, can provide a visualization of the classification of the data, which is very useful, in general, for quickly inspecting the success of the classification. The obtained LDA scatter plot is illustrated in Figure 1, where the circles denote the LIBS spectra used for the training of the algorithm, while the same color (light) stars denote the LIBS spectra used for testing. Throughout this study, the data points of pure EVOOs’ are designated with a grey color, while each type of adulterated mixture is designated with a different color, according to the color code indicated in Figure 1. As can be seen, the class of EVOOs is visibly well separated from the classes of the adulterated samples, with the test data points (i.e., the stars) being well-fitted within the class. However, regarding the adulterated EVOO samples, some limited overlapping occurs between the four classes of adulterants, as can be seen in Figure 1.

The classification accuracy, obtained via a 10-fold cross-validation process, was determined to be (84.9 ± 1.5)%, while the predictive accuracy, estimated via external validation, was 87.6% (see also Table 2). The classification results of the unknown LIBS spectra are also shown, in more detail, in the corresponding confusion matrix, as presented in Table 2. As can be seen, the LDA model has successfully predicted all 240 spectra of the EVOOs used for testing, while it has falsely classified various spectra of their adulterated mixtures.

Next, an analysis of the LIBS spectra via the SVM, the LR, and the GB algorithms was performed. The obtained results were also successful, with the SVM reaching a classification accuracy of (88.5 ± 1.2%) and a high predictive accuracy of 92.2%, the LR algorithm attaining a classification accuracy of (85.9 ± 1.3%) and a predictive accuracy of 89.1%, and the GB algorithm achieving a classification accuracy of (88.8 ± 1.3%) and a predictive accuracy of 88.1% (see also Table 2). For a better insight into the performance of each algorithm, the corresponding confusion matrices were constructed; they are all illustrated in Table S1. It is worth noting that the majority of LIBS spectra were correctly classified.

The above findings demonstrate that the LIBS technique combined with machine learning can very successfully discriminate pure EVOOs from adulterated ones. All statistical models applied managed to discriminate the EVOOs from the adulterated ones and classify them correctly, in the respective classes, achieving excellent predictive accuracies. Concerning the identification of the adulterant used, successful predictive accuracies were obtained as well, although slightly lower, compared to the previous analysis. To shed more light on the aspects of identifying the type of adulterant, the EVOOs from each geographical region (Crete, Lesvos, Kalamata, and Achaia) and their mixtures were analyzed, and the obtained results are presented and discussed below.

### 2.3. Analysis of EVOOs from Different Geographical Regions and Their Mixtures with Lower-Quality Oils

In this view, the LIBS spectroscopic data of EVOOs and the mixtures of each region were initially pretreated by means of the PCA algorithm. Again, the optimum number of PCs for all the constructed predictive models was searched; it was found that 80, 50, 110, and 40 PCs were adequate for the EVOOs from Crete, Lesvos, Kalamata, and Achaia, respectively.

**Figure 2:**
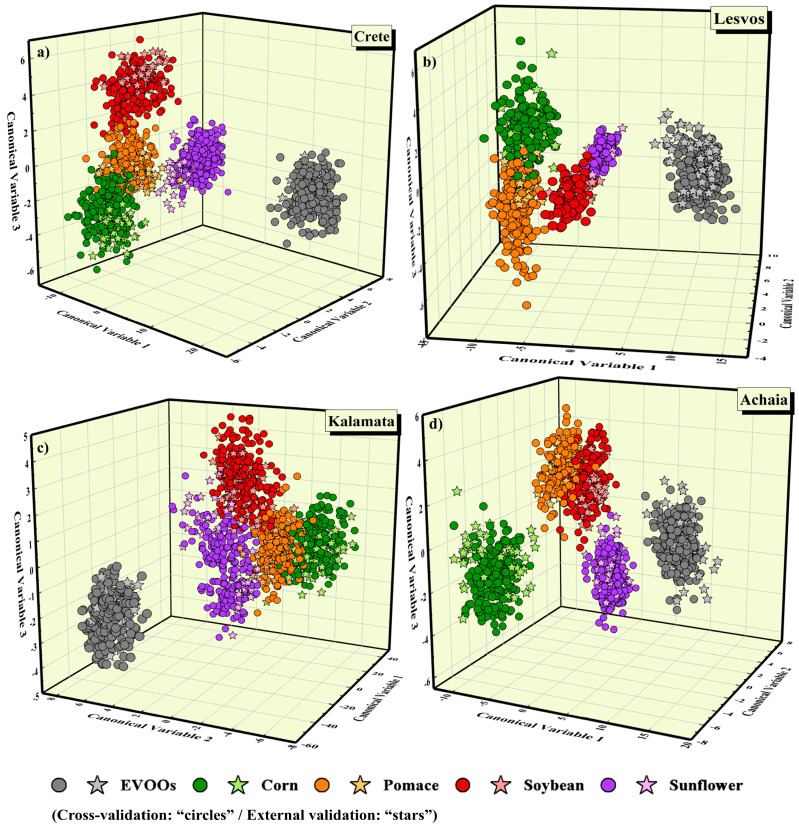
LDA plots showing the classification of EVOOs from (**a**) Crete, (**b**) Lesvos, (**c**) Kalamata, and (**d**) Achaia, and their mixtures with corn, pomace, soybean, and sunflower oils.

Then, the preprocessed data of each region were analyzed using the LDA algorithm; the corresponding LDA scatter plots are presented in Figure 2. As shown, in all cases, the class of pure EVOOs was clearly separated from the four classes of adulterated samples. In addition, the EVOOs’ test data points (i.e., stars) were also well fitted in their respective class. As for the mixtures, in all cases, the four classes were found to be well formed, while minor overlapping and/or the dispersion of various data points was observed. In particular, the test data points of EVOOs from Crete and Kalamata mixed with sunflower oil showed some dispersion (see Figure 2a,c). Moreover, for the EVOOs from Lesvos, some overlapping occurred between their mixtures with corn and pomace oils and between their mixtures with soybean and sunflower oils (see Figure 2b). As for the EVOOs from Achaia, overlapping between the mixtures with pomace and soybean oils was observed (see Figure 2d). A better view of the model’s performance can be found in the corresponding confusion matrices, as presented in Table 3. As can be seen, the classification results were successful, as all the EVOOs’ LIBS spectra were correctly predicted, and only some LIBS spectra of mixtures were misclassified.

The classification and predictive accuracies attained using the LDA algorithm were remarkable since, for every geographical region, values reaching almost 99% were obtained. In more detail, classification accuracies of (98.5 ± 1.4%), (96.8 ± 1.5%), (92.9 ± 2.6%), and (97.0 ± 2.1%) were yielded for the EVOOs from Crete, Lesvos, Kalamata, and Achaia, respectively. More importantly, extremely high predictive accuracies were attained, i.e., 99.3%, 95.0%, 94.3%, and 98.3%, for the EVOOs from Crete, Lesvos, Kalamata, and Achaia, respectively (see Table 3).

Subsequently, the SVM, LR, and GB algorithms were applied, and the corresponding predictive models were constructed and tested. Similarly successful results were reached (see Table 3). Thus, in the case of EVOOs from Crete, the SVM, LR, and GB algorithms achieved classification accuracies of (98.4 ± 0.7%), (98.4 ± 1.2%), and (94.1 ± 1.3%), and predictive accuracies of 98.7%, 99.3%, and 91.3%, respectively. Concerning the EVOOs from Lesvos, the SVM, LR, and GB algorithms resulted in classification accuracies of (97.9 ± 1.2%), (98.1 ± 1.4%), and (95.3 ± 2.4%) and predictive accuracies of 96.0%, 97.3%, and 94.0%, respectively. For the EVOOs from Kalamata, the corresponding classification accuracies achieved using the SVM, LR, and GB algorithms were (93.7 ± 1.4%), (93.3 ± 1.5%), and (88.1 ± 3.4%) and the predictive accuracies were 95.0%, 94.6%, and 88.3%, respectively. As for the EVOOs from Achaia, classification accuracies of (96.7 ± 1.9%), (95.0 ± 2.4%), and (90.4 ± 2.4%) and predictive accuracies of 96.0%, 95.3%, and 88.6% were obtained, respectively, for the SVM, LR, and GB algorithms. A better insight into the classification results for these models is provided by the confusion matrices presented in Table S1. As shown, the SVM and LR algorithms correctly classified almost all spectra, while the GB algorithm misclassified a few LIBS spectra. Overall, the classification results are considered very successful.

From a comparison of the models’ performances, it can be observed that the LDA, SVM, and LR algorithms were found to be the most efficient for identifying the type of adulterant, yielding the highest accuracies, with the GB algorithm attaining slightly lower values in all cases. This outcome might be due to the principles of the LDA, SVM, and LR algorithms, all employing a function for classification analysis, while the GB algorithm employs decision trees. This observation might imply some linear correlations between the LIBS spectroscopic data.

## 3. Materials and Methods

### 3.1. EVOOs, Adulterants, and Datasets

In the present study, 40 Greek extra virgin olive oil samples (EVOOs) were collected from local producers in Crete, Lesvos, Kalamata, and Achaia (10 samples from each region) and were studied. One sample from each region was used for the preparation of binary mixtures with commercially available soybean, sunflower, corn, and pomace oil (purchased from a local market). The binary mixtures were prepared with various mixing ratios, i.e., from 10 to 90% *w*/*w*, with a step of 10%, resulting in nine different concentrations for each adulterant employed. A detailed description of the studied samples is presented in Table S2. All samples were kept in dark glass bottles and stored in a refrigerator at a temperature of −2 to −4 °C; prior to measurements, they were left at room temperature for a few hours. A quantity of 1.5–2 mL of each sample, placed in a shallow glass recipient, was used for the LIBS measurements.

For the statistical analysis, at first, all EVOOs were considered as one class (i.e., 40 samples), while all the adulterated samples were considered as a second class (i.e., 144 mixtures). Therefore, in this case, 184 samples in total were examined. From these samples, 144 mixtures were used for training the algorithmic models (i.e., 32 EVOOs and 112 mixtures), and 40 samples (i.e., 8 EVOOs and 32 mixtures) were used for testing.

In the second approach, for the identification of the type of adulterant, the 184 samples (i.e., the 40 EVOOs and the 144 mixtures) were considered as five classes, i.e., one comprising EVOOs and four classes comprising the pomace, corn, soybean, and sunflower mixtures, respectively. From these samples, 32 EVOOs, 28 adulterated with corn, 28 with pomace, 28 with soybean, and 28 with sunflower (in total 144 samples) were used for training, while 8 EVOOs, 8 adulterated with corn, 8 with pomace, 8 with soybean, and 8 with sunflower (i.e., in total 40) were used for testing (see Table S3). For a better insight into this approach, EVOOs and mixtures from each different region were also considered separately. In this case, 8 EVOOs, 7 adulterated with corn, 7 with pomace, 7 with soybean, and 7 with sunflower (in total 36 samples) were used for training, while 2 EVOOs, 2 adulterated with corn, 2 with pomace, 2 with soybean, and 2 with sunflower (in total 10 samples) were used for testing.

### 3.2. Experimental Setup

For the LIBS measurements, the 1064 nm output from a 5 ns Q-switched Nd:YAG laser (Quanta-Ray INDI, Spectra Physics, Santa Clara, CA, USA), operating at a repetition rate of 3 Hz, was used. The laser beam was incident vertically on the samples’ surface, and it was focused via a 150 mm lens. The laser beam energy was about 80 mJ. A steady flow of Argon gas above the surface of the samples was applied to minimize splashing from the samples. The light emitted from the plasma created on the samples’ surface was collected by means of a 50 mm lens and was introduced into a quartz optical fiber coupled to the entrance slit (10 μm) of a portable spectrograph (AvaSpec-ULS4096CL-EVO, Avantes, Apeldoorn, The Netherlands), equipped with a 300-line/mm grating and a 4096-pixel CMOS detector. From these pixels, 2751 were utilized, corresponding to a spectral range from 200 to 1000 nm. As for the gating conditions of the detector, a time delay (t_d_) of 1.28 μs and an integration time (t_w_) of 1.05 ms were employed. The LIBS spectra from 10 consecutive laser shots were averaged, corresponding to a single LIBS measurement, while 30 such LIBS measurements were obtained from different places on the samples’ surface to account for any inhomogeneity and better sampling to ensure the reliability of the collected spectral data for the subsequent statistical analysis. 

### 3.3. LIBS Spectra of Oils

**Figure 3:**
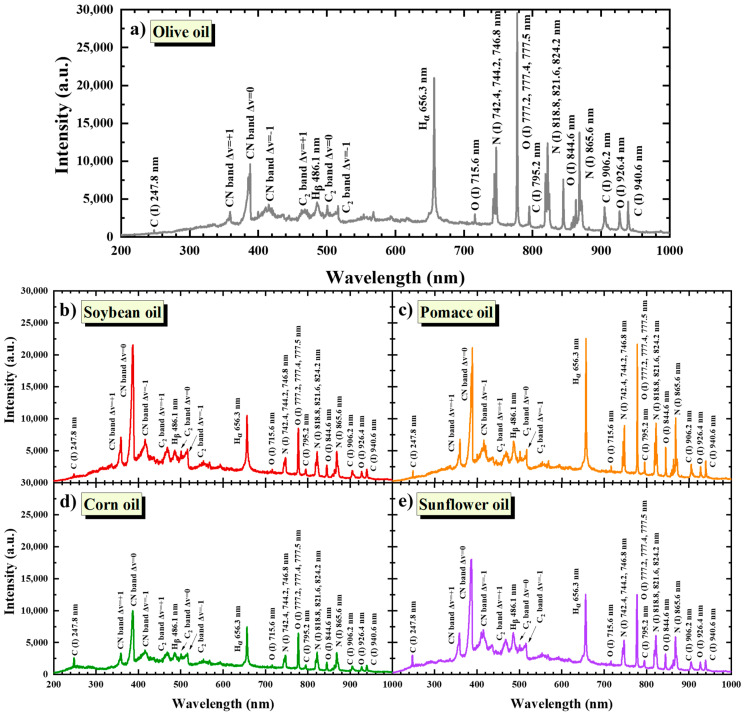
LIBS spectra of (**a**) olive oil, (**b**) soybean oil, (**c**) pomace oil, (**d**) corn oil, and (**e**) sunflower oil.

Olive oil and other edible oils, including soybean, pomace, corn, and sunflower oils, consist mainly of fatty acids (saturated, mono-, and poly-unsaturated ones), while polyphenols, carotenoids, chlorophyll, pigments, vitamins, etc., are also present in smaller amounts. Since the elemental composition of the constituents of these oils comprises mainly carbon (C), oxygen (O), hydrogen (H), and nitrogen (N) atoms, the LIBS spectra of these oils exhibit the characteristic spectral features of these elements and are very similar. This is shown in Figure 3, where various representative LIBS spectra of olive oil, soybean, pomace, corn, and sunflower oils are presented. 

As can be seen, the atomic emissions—C(I) at 247.8, 795.2, 906.2, and 940.6 nm; O(I) at 715.6, 777.2, 777.4, 777.5, 844.6, and 926.4 nm; Ha at 656.3 nm; Hβ at 486.1 nm; and N(I) at 742.4, 744.2, 746.8, 818.8, 821.6, 824.2, 862.9, and 865.6 nm—are readily observable in all cases, together with the cyanogen (CN) and C2 molecular bands. In particular, the vibrational progression of the CN band is observed at 360, 388, and 422 nm (Δv = +1, 0, −1), while the C2 Swan band system is observed at 470, 516, and 559 nm (Δv = +1, 0, −1). The assignment of the plasma emissions was based on the National Institute of Standards and Technology (NIST) atomic spectra database [25]. Interestingly, the relative intensities of the above-mentioned spectral features appearing in the LIBS spectra of Figure 3a–e are significantly different for the different oils, with the olive oil, followed by pomace oil, exhibiting the strongest spectral features and corn oil exhibiting the lowest ones.

### 3.4. Data Analysis 

To analyze the spectroscopic data, different machine learning algorithms were employed, i.e., Principal Component Analysis (PCA), Linear Discriminant Analysis (LDA), Support Vector Machines (SVMs), Logistic Regression (LR), and Gradient Boosting (GB). The implementation of the algorithms was performed in a Python programming environment, employing the Scikit-Learn library and the XGBoost library for the GB algorithm. A brief description of the principles of these algorithms can be found elsewhere [26]. The LIBS spectroscopic data were scaled by using the standard scaler (in Python programming environment), i.e., the mean of each feature was subtracted, and the values were then scaled to unit variance. It should be noted that no further preprocessing was applied to the data.

For all the algorithms employed, the corresponding predictive models were constructed and their performance was accessed by means of the “k-fold” cross-validation method [27]. According to this method, the initial dataset is divided into k subsets (k = 10 in this study). During each iteration, k − 1 subsets are employed for training the algorithm, and the k subset left out is used for evaluating the models’ efficiency. Moreover, to further ensure the robustness of the prepared models, an external validation procedure is conducted, according to which a specific number of samples is entirely excluded from the training process and used only for prediction purposes. At this point, it should be mentioned that during the cross-validation, the k − 1 subsets of the original correlated data are transformed using the PCA algorithm to a new dimensional space (i.e., that of the PCs). The remaining single subset is then projected to the PCs’ space. This procedure is repeated for each fold (i.e., k times). Similarly, this happens during the external validation. For each fold, scaling of the data is applied as well.

In addition, the corresponding confusion matrices were also generated, which contain rows and columns, with the former corresponding to the actual classes and the latter corresponding to the predicted classes. The diagonal elements of such matrices depict the correct predictions (i.e., the number of correctly classified LIBS spectra), while the non-diagonal elements show the incorrect ones (i.e., the misclassified LIBS spectra).

## 4. Conclusions

In the present work, the detection of the adulteration of olive oil samples using the LIBS technique employing machine learning algorithms was investigated. The different issues of the discrimination of a pure EVOO sample from an adulterated one and the identification of the adulterant were addressed. To accomplish these tasks, EVOOs from different regions of Greece, namely, Crete, Lesvos, Kalamata, and Achaia, were used, and various commercial lower-quality oils, namely, pomace, soybean, corn, and sunflower, were used to prepare binary/adulterated mixtures. The LIBS spectroscopic data were analyzed by employing several machine learning algorithms, i.e., PCA, LDA, SVMs, LR, and GB, which were evaluated systematically for their performance using both internal and external validation procedures. For the discrimination of the EVOO samples from the adulterated samples, all statistical models were found to operate successfully, achieving excellent predictive accuracies, attaining values as high as almost 100%. For the identification of the adulterant, accuracies between 92% and 99% were obtained. The present results unambiguously demonstrate the potential of LIBS for the detection of olive oil adulteration and its eventual implementation as an effective tool for fraud traceability, ensuring transparency in the supply chain and, consequently, the quality of olive oils.

## Figures and Tables

**Table 1:** Confusion matrices, classification, and prediction accuracies obtained using the different models (i.e., LDA, SVMs, LR, and GB) for all EVOOs and their mixtures with lower-quality oils (i.e., corn, pomace, soybean, sunflower).

		**LDA**		**SVMs**		**LR**		**GB**	
**Classification accuracy (%)**		100.0 ± 0.0		100.0 ± 0.0		100.0 ± 0.0		99.7 ± 0.1	
**Predictive accuracy (%)**		100		100		100		99.8	
		**Predicted classes**							
		**EVOOs**	**Mixtures**	**EVOOs**	**Mixtures**	**EVOOs**	**Mixtures**	**EVOOs**	**Mixtures**
**Actual classes**	**EVOOs**	240	0	240	0	240	0	238	2
	**Mixtures**	0	960	0	960	0	960	1	959

**Table 2:** Classification and predictive accuracies obtained using each model for all EVOOs and their mixtures and confusion matrix for the external validation procedure using LDA.

			**LDA**	**SVMs**	**LR**	**GB**
**Classification accuracy (%)**			84.9 ± 1.5	88.5 ± 1.2	85.9 ± 1.3	88.8 ± 1.3
**Predictive accuracy (%)**			87.6	92.2	89.1	88.1
		**Predicted classes**				
		**EVOOs**	**Corn oil**	**Pomace oil**	**Soybean oil**	**Sunflower oil**
**Actual classes**	**EVOOs**	**240**	0	0	0	0
	**Corn oil**	0	**193**	32	13	2
	**Pomace oil**	0	19	**203**	15	3
	**Soybean oil**	0	3	16	**202**	19
	**Sunflower oil**	0	4	3	20	**213**

**Table 3:** Classification and predictive accuracies obtained using each model for EVOOs from each region and their mixtures and confusion matrix for the external validation procedure using LDA.

**Geographical Origin**				**LDA**	**SVMs**	**LR**	**GB**
**Crete**	**Classification accuracy (%)**			98.5 ± 1.4	98.4 ± 0.7	98.4 ± 1.2	94.1 ± 1.3
	**Predictive accuracy (%)**			99.3	98.7	99.3	91.3
**Lesvos**	**Classification accuracy (%)**			96.8 ± 1.5	97.9 ± 1.2	98.1 ± 1.4	95.3 ± 2.4
	**Predictive accuracy (%)**			95.0	96.0	97.3	94.0
**Kalamata**	**Classification accuracy (%)**			92.9 ± 2.6	93.7 ± 1.4	93.3 ± 1.5	88.1 ± 3.4
	**Predictive accuracy (%)**			94.3	95.0	94.6	88.3
**Achaia**	**Classification accuracy (%)**			97.0 ± 2.1	96.7 ± 1.9	95.0 ± 2.4	90.4 ± 2.4
	**Predictive accuracy (%)**			98.3	96.0	95.3	88.6
**Geographical origin**			**Predicted classes**				
			**EVOOs**	**Corn oil**	**Pomace oil**	**Soybean oil**	**Sunflower oil**
**Crete**	**Actual classes**	**EVOOs**	**60**	0	0	0	0
		**Corn oil**	0	**60**	0	0	0
		**Pomace oil**	0	0	**59**	0	1
		**Soybean oil**	0	0	0	**60**	0
		**Sunflower oil**	0	0	0	1	**59**
**Lesvos**	**Actual classes**	**EVOOs**	**60**	0	0	0	0
		**Corn oil**	0	**60**	0	0	0
		**Pomace oil**	0	12	**48**	0	0
		**Soybean oil**	0	0	0	**60**	0
		**Sunflower oil**	0	0	0	3	**57**
**Kalamata**	**Actual classes**	**EVOOs**	**60**	0	0	0	0
		**Corn oil**	0	**56**	4	0	0
		**Pomace oil**	0	1	**59**	0	0
		**Soybean oil**	0	0	2	**56**	2
		**Sunflower oil**	0	0	0	8	**52**
**Achaia**	**Actual classes**	**EVOOs**	**60**	0	0	0	0
		**Corn oil**	0	**60**	0	0	0
		**Pomace oil**	0	0	**59**	1	0
		**Soybean oil**	0	0	2	**58**	0
		**Sunflower oil**	0	0	0	2	**58**

## Data Availability

Data will be made available upon request.

## Supplementary Materials

The supporting information can be downloaded at: https://www.mdpi.com/article/10.3390/molecules28247960/s1. Table S1. Confusion matrices obtained via external validation using the SVM/LR/GB algorithms; Table S2. Number of EVOOs per geographical region and mixtures with lower-quality oils; Table S3. Number of samples/spectra used for training and testing per geographical region and mixtures.

## References

[1] Bungaro M. The World of Olive Oil. https://www.internationaloliveoil.org/the-world-of-olive-oil/.

[2] Olive Oil. https://www.internationaloliveoil.org/olive-world/olive-oil/.

[3] Dashboard: Olive Oil | Agriculture and Rural Development. https://agriculture.ec.europa.eu/document/306cf510-5934-4488-b9c1-d6abf264c381_en.

[4] Geographical Indications and Quality Schemes Explained. https://agriculture.ec.europa.eu/farming/geographical-indications-and-quality-schemes/geographical-indications-and-quality-schemes-explained_en.

[5] Georgouli K., Martinez Del Rincon J., Koidis A. (2017). Continuous Statistical Modelling for Rapid Detection of Adulteration of Extra Virgin Olive Oil Using Mid Infrared and Raman Spectroscopic Data. Food Chem..

[6] Mu T., Chen S., Zhang Y., Chen H., Guo P., Meng F. (2016). Portable Detection and Quantification of Olive Oil Adulteration by 473-Nm Laser-Induced Fluorescence. Food Anal. Methods.

[7] Meng X., Yin C., Yuan L., Zhang Y., Ju Y., Xin K., Chen W., Lv K., Hu L. (2023). Rapid Detection of Adulteration of Olive Oil with Soybean Oil Combined with Chemometrics by Fourier Transform Infrared, Visible-near-Infrared and Excitation-Emission Matrix Fluorescence Spectroscopy: A Comparative Study. Food Chem..

[8] Miziolek A.W., Palleschi V., Schechter I. (2006). Laser-Induced Breakdown Spectroscopy (LIBS): Fundamentals and Applications.

[9] Noll R. (2012). Laser-Induced Breakdown Spectroscopy.

[10] Noll R., Bette H., Brysch A., Kraushaar M., Mönch I., Peter L., Sturm V. (2001). Laser-Induced Breakdown Spectrometry—Applications for Production Control and Quality Assurance in the Steel Industry. Spectrochim. Acta Part B Spectrosc..

[11] Gazeli O., Stefas D., Couris S. (2021). Sulfur Detection in Soil by Laser Induced Breakdown Spectroscopy Assisted by Multivariate Analysis. Materials.

[12] Stefas D., Gyftokostas N., Bellou E., Couris S. (2019). Laser-Induced Breakdown Spectroscopy Assisted by Machine Learning for Plastics/Polymers Identification. Atoms.

[13] Anglos D., Couris S., Fotakis C. (1997). Laser Diagnostics of Painted Artworks: Laser-Induced Breakdown Spectroscopy in Pigment Identification. Appl. Spectrosc..

[14] Gaudiuso R., Dell’Aglio M., Pascale O.D., Senesi G.S., Giacomo A.D. (2010). Laser Induced Breakdown Spectroscopy for Elemental Analysis in Environmental, Cultural Heritage and Space Applications: A Review of Methods and Results. Sensors.

[15] Sezer B., Bilge G., Boyaci I.H. (2017). Capabilities and Limitations of LIBS in Food Analysis. Trends Anal. Chem..

[16] Stefas D., Gyftokostas N., Nanou E., Kourelias P., Couris S. (2021). Laser-Induced Breakdown Spectroscopy: An Efficient Tool for Food Science and Technology (from the Analysis of Martian Rocks to the Analysis of Olive Oil, Honey, Milk, and Other Natural Earth Products). Molecules.

[17] Caceres J.O., Moncayo S., Rosales J.D., De Villena F.J.M., Alvira F.C., Bilmes G.M. (2013). Application of Laser-Induced Breakdown Spectroscopy (LIBS) and Neural Networks to Olive Oils Analysis. Appl. Spectrosc..

[18] Bellou E., Gyftokostas N., Stefas D., Gazeli O., Couris S. (2020). Laser-Induced Breakdown Spectroscopy Assisted by Machine Learning for Olive Oils Classification: The Effect of the Experimental Parameters. Spectrochim. Acta Part B Spectrosc..

[19] Kongbonga Y.G.M., Ghalila H., Onana M.B., Ben Lakhdar Z. (2014). Classification of Vegetable Oils Based on Their Concentration of Saturated Fatty Acids Using Laser Induced Breakdown Spectroscopy (LIBS). Food Chem..

[20] Gazeli O., Bellou E., Stefas D., Couris S. (2020). Laser-Based Classification of Olive Oils Assisted by Machine Learning. Food Chem..

[21] Gyftokostas N., Stefas D., Couris S. (2020). Olive Oils Classification via Laser-Induced Breakdown Spectroscopy. Appl. Sci..

[22] Gyftokostas N., Stefas D., Kokkinos V., Bouras C., Couris S. (2021). Laser-Induced Breakdown Spectroscopy Coupled with Machine Learning as a Tool for Olive Oil Authenticity and Geographic Discrimination. Sci. Rep..

[23] Gyftokostas N., Nanou E., Stefas D., Kokkinos V., Bouras C., Couris S. (2021). Classification of Greek Olive Oils from Different Regions by Machine Learning-Aided Laser-Induced Breakdown Spectroscopy and Absorption Spectroscopy. Molecules.

[24] Stefas D., Gyftokostas N., Kourelias P., Nanou E., Kokkinos V., Bouras C., Couris S. (2021). Discrimination of Olive Oils Based on the Olive Cultivar Origin by Machine Learning Employing the Fusion of Emission and Absorption Spectroscopic Data. Food Control.

[25] Kramida A., Ralchenko Y. (2022). NIST Atomic Spectra Database, NIST Standard Reference Database 78 [Data Set].

[26] Nanou E. (2023). Identification of the Animal Origin of Milk via Laser-Induced Breakdown Spectroscopy. Food Control.

[27] Berrar D. (2018). Cross-Validation. Encyclopedia of Bioinformatics and Computational Biology.

