# Supplementary material for: Rapid Authentication and Detection of Olive Oil Adulteration Using Laser-Induced Breakdown Spectroscopy
Source: Molecules. 2023 Dec 5;28(24):7960. doi: 10.3390/molecules28247960 (PMC10745825; doi:10.3390/molecules28247960)
Supplement: Supplementary file 1 [file molecules-28-07960-s001.zip › molecules-2731334-supplementary.pdf]

Article

Rapid Authentication and Detection of Olive Oil Adulteration using Laser-Induced Breakdown Spectroscopy

Eleni Nanou [1,2], Nefeli Pliatsika [1,2] and Stelios Couris [1,2,*]

[1] Department of Physics, University of Patras, 26504 Patras, Greece; e.nanou@iceht.forth.gr (E.N.); n.pliatsika@iceht.forth.gr (N.P.)
[2] Institute of Chemical Engineering Sciences (ICE-HT), Foundation for Research and Technology-Hellas (FORTH), 26504 Patras, Greece
* Correspondence: couris@upatras.gr; Tel.: +30-2610996086

Table S1. Confusion matrices obtained via external validation by the SVMs/LR/GB algorithms.

SVMs/LR/GB algorithms							
**Geographical origin**			**Predicted classes**				
			**EVOOs**	**Corn oil**	**Pomace oil**	**Soybean oil**	**Sunflower oil**
**All regions**	**Actual classes**	**EVOOs**	**240/240/238**	0/0/0	0/0/2	0/0/0	0/0/0
		**Corn oil**	0/0/0	**218/199/211**	21/31/17	1/7/8	0/3/4
		**Pomace oil**	0/0/0	12/20/19	**214/206/203**	8/11/7	6/3/11
		**Soybean oil**	0/0/1	4/8/1	11/16/15	**218/200/205**	7/16/18
		**Sunflower oil**	0/0/0	5/1/3	2/1/7	17/14/30	**216/224/200**
**Crete**	**Actual classes**	**EVOOs**	**60/60/56**	0/0/0	0/0/0	0/0/3	0/0/1
		**Corn oil**	0/0/0	**60/60/53**	0/0/0	0/0/0	0/0/7
		**Pomace oil**	0/0/0	0/0/5	**59/59/52**	0/0/1	1/1/2
		**Soybean oil**	0/0/0	0/0/0	0/0/0	**60/60**	0/0/0
		**Sunflower oil**	0/0/0	0/0/3	3/1/0	0/0/a	**57/59/53**
**Lesvos**	**Actual classes**	**EVOOs**	**60/60/59**	0/0/1	0/0/0	0/0/0	0/0/0
		**Corn oil**	1/0/0	**57/57/52**	2/3/8	0/0/0	0/0/0
		**Pomace oil**	0/0/0	1/1/4	**59/59/56**	0/0/0	0/0/0
		**Soybean oil**	0/0/0	0/0/0	0/0/0	**59/60/59**	1/0/1
		**Sunflower oil**	0/0/0	0/0/0	0/0/0	7/4/4	**53/56/56**
**Kalamata**	**Actual classes**	**EVOOs**	**60/60/57**	0/0/3	0/0/0	0/0/0	0/0/0
		**Corn oil**	0/0/0	**56/56/54**	4/3/6	0/0/0	0/1/0
		**Pomace oil**	0/0/0	5/0/4	**55/59/52**	0/1/1	0/0/3

		**Soybean oil**	0/0/0	0/0/0	0/0/2	**58/56/54**	2/4/4
		**Sunflower oil**	0/0/0	0/0/3	1/0/1	3/7/8	**56/53/48**
**Achaia**	**Actual classes**	**EVOOs**	**60/60/59**	0/0/0	0/0/0	0/0/0	0/0/1
		**Corn oil**	0/0/1	**60/60/55**	0/0/4	0/0/0	0/0/0
		**Pomace oil**	0/0/0	0/0/0	**55/53/51**	5/7/9	0/0/0
		**Soybean oil**	0/0/2	0/0/1	2/3/9	**55/55/43**	3/2/5
		**Sunflower oil**	0/0/0	0/0/0	1/0/0	1/2/2	**58/58/58**

Table S2. Number of EVOOs per geographical region and mixtures with lower quality oils.

Geographical origin	EVOOs	Adulterant	No. of Adulteration samples	Total No. of samples
**Crete**	10	Corn<br>Pomace<br>Soybean<br>Sunflower	9<br>9<br>9<br>9	46
**Lesvos**	10	Corn<br>Pomace<br>Soybean<br>Sunflower	9<br>9<br>9<br>9	46
**Kalamata**	10	Corn<br>Pomace<br>Soybean<br>Sunflower	9<br>9<br>9<br>9	46
**Achaia**	10	Corn<br>Pomace<br>Soybean<br>Sunflower	9<br>9<br>9<br>9	46
**All regions**	40	Corn, Pomace, Soybean, Sunflower	144	184

Table S3. Number of samples/spectra used for training and testing, per geographical region and mixtures.

Geographical origin	Classes	Training Samples	Measurements	Training Spectra	Testing Samples	Measurements	Testing Spectra
**Crete**	EVOOs	8	30	240	2	30	60
	Corn	7	30	210	2	30	60
	Pomace	7	30	210	2	30	60
	Soybean	7	30	210	2	30	60
	Sunflower	7	30	210	2	30	60
**Lesvos**	EVOOs	8	30	240	2	30	60
	Corn	7	30	210	2	30	60
	Pomace	7	30	210	2	30	60
	Soybean	7	30	210	2	30	60
	Sunflower	7	30	210	2	30	60
**Kalamata**	EVOOs	8	30	240	2	30	60
	Corn	7	30	210	2	30	60
	Pomace	7	30	210	2	30	60
	Soybean	7	30	210	2	30	60
	Sunflower	7	30	210	2	30	60
**Achaia**	EVOOs	8	30	240	2	30	60
	Corn	7	30	210	2	30	60
	Pomace	7	30	210	2	30	60
	Soybean	7	30	210	2	30	60
	Sunflower	7	30	210	2	30	60
**All regions**	EVOOs	32	30	960	8	30	240
	Corn	28	30	840	8	30	240
	Pomace	28	30	840	8	30	240
	Soybean	28	30	840	8	30	240
	Sunflower	28	30	840	8	30	240
